# Activation of bisulfite by LaFeO_3_ loaded on red mud for degradation of organic dye

**DOI:** 10.1098/rsos.220466

**Published:** 2022-11-30

**Authors:** Yao Li, Xiangyu Meng, Yin Pang, Cong Zhao, Daoping Peng, Yu Wei, Bayongzhong Xiang

**Affiliations:** ^1^ Faculty of Geosciences and Environmental Engineering, Southwest Jiaotong University, Chengdu 610031, People's Republic of China; ^2^ State Key Laboratory of Materials Processing and Die and Mould Technology, School of Materials Science and Technology, Huazhong University of Science and Technology, Wuhan 430074, People's Republic of China

**Keywords:** red mud, perovskite, sulfate radical, fenton-like, dyes, waste utilization

## Abstract

In this study, red mud (RM) was used as a support for LaFeO_3_ to prepare LaFeO_3_-RM via the ultrasonic-assisted sol–gel method for the removal of methylene blue (MB) assisted with bisulfite (BS) in the aqueous solution. Characterization by scanning electron microscopy and the Brunauer–Emmett–Teller method indicated that LaFeO_3_-RM exhibited a large surface area and porous structure with a higher pore volume (i.e. 10 times) compared with the bulk LaFeO_3_. The XRD, XPS and FTIR results revealed that the support of porous RM not only dispersed LaFeO_3_ particles but also increased Fe oxidation capability, oxygen-containing functional groups and chemically adsorbed oxygen (from 44.3% to 90.3%) of LaFeO_3_-RM, which improved the catalytic performance in structure and chemical composition. MB was removed through the synergistic effect of adsorption and catalysis, with MB molecules first absorbed on the surface and then degraded. The removal efficiency was 88.19% in the LaFeO_3_-RM/BS system under neutral conditions but only 27.09% in the LaFeO_3_/BS system. The pseudo-first-order kinetic constant of LaFeO_3_-RM was six times higher than that of LaFeO_3_. Fe(III) in LaFeO_3_-RM played a key role in the activation of BS to produce ${\mathrm{SO}}_{4}\cdot^{-}$ by the redox cycle of Fe(III)/Fe(II). Dissolved oxygen was an essential factor for the generation of ${\mathrm{SO}}_{4}\cdot^{-}$. This work provides both a new approach for using porous industrial waste to improve the catalytic performance of LaFeO_3_ and guidance for resource utilization of RM in wastewater treatment.

## 1. Introduction

Dye wastewater from various industries has posed a serious threat to the ecological environment and human health due to its high toxicity and non-biodegradability [1]. Methylene blue (MB), as an artificial organic cationic dye, is widely used as a colouring agent and redox indicator [2,3]. However, the aromatic rings in it could cause carcinogenic and mutagenic effects [4]. Therefore, developing effective treatment of dye wastewater is of vital importance.

The current treatment methods for dye wastewater include adsorption [5,6], catalytic ozonation [7], membrane separation [8] and advanced oxidation processes (AOPs) [9–11]. Among them, AOPs based on sulfate radical (${\mathrm{SO}}_{4}\cdot^{-}$) are widely used to remove organic pollutants in wastewater [12]. Compared with OH·, ${\mathrm{SO}}_{4}\cdot^{-}$ has a higher redox potential (*E*^0^ = 2.5–3.1 V) [13] and can degrade pollutants in a wider pH range (4 < pH < 9) [14]. Normally, ${\mathrm{SO}}_{4}\cdot^{-}$ is produced through activation of persulfate (PS) by UV [15], heat [16], ultrasound [17] and transition metals [18–20]. However, the application of PS was limited due to its slow reaction rate, secondary pollution and low stability in water [13]. Therefore, bisulfite (BS) is attracting increasing attention for its low cost and environment-friendly characteristics. Recently, studies have found that BS can also be activated by UV [21] and transition metals [22,23] to produce ${\mathrm{SO}}_{4}\cdot^{-}$. However, the application of the BS activation system is limited due to its low efficiency. Therefore, searching for an effective BS activation system has attracted increasing interest.

Recently, different heterogeneous iron-based catalysts have been widely studied in AOPs for their high catalytic activity and natural abundances [24–26]. LaFeO_3_, as an iron-based compound, has shown catalytic performance in the activation of PS. [27]. As an ABO_3_ perovskite oxide with Fe element in B sites, LaFeO_3_ could be used for activating PS through the redox cycle of Fe(III)/Fe(II) [28]. LaFeO_3_ shows higher catalytic activity than Fe_2_O_3_ in degrading diclofenac by activating PMS [29]. Therefore, LaFeO_3_ shows great potential for BS activation.

However, due to the high temperature required in the synthesis of LaFeO_3_, bulk LaFeO_3_ was non-porous and its specific surface area was usually below 10 m^2^ g^−1^, resulting in low catalytic activity [30]. Therefore, in order to improve the catalytic activity of LaFeO_3_, LaFeO_3_ was supported on porous supports to obtain supported perovskite catalysts with increased surface areas and porous structures. SiO_2_ [31,32] and Al_2_O_3_ [33] were found to be effective supports for LaFeO_3_. It is interesting to load LaFeO_3_ on porous supports due to their adsorption capacity, which allows the pollutants to be adsorbed on the surface and then degraded [32]. Therefore, the supported LaFeO_3_ catalysts could realize the synergistic effect of adsorption and catalysis. However, those supports used in previous studies are costly. Consequently, it is of vital importance to find cheap and easily available supports. A previous study suggested porous materials with randomly distributed pores and short pore lengths were more suitable as supports for LaFeO_3_ [31].

Red mud (RM) was considered a suitable support for LaFeO_3_ due to its large surface area, abundant randomly distributed pores and high stability in water [34]. As a typical solid waste produced in alumina production using the Bayer process, the production of RM reached 77 million tons globally in 2017 [35,36]. As a cost-free, environmentally friendly and functional material with application prospects, RM contains many residual bauxite minerals, including hematite, magnetite and titanium dioxide, which could be used as catalysts or adsorbents in dye water treatment [37]. The rich Fe_2_O_3_ (15.2%–62.8%) contained in RM makes it an ideal catalyst for the activation of BS [38,39]. Effective utilization of RM can reduce the landfill of RM, which can cause damage to the environment [40]. Therefore, loading LaFeO_3_ on RM could be an effective and environmentally friendly way to improve the catalytic performance of bulk LaFeO_3_. Simultaneously, the reduction and waste utilization of RM were also achieved in this way. In addition, this type of supported perovskite catalysts for BS activation has not been reported.

In this study, LaFeO_3_-RM was successfully synthesized using the ultrasonic-assisted sol–gel method. To understand the mechanism of the LaFeO_3_-RM/BS system on MB removal, the objectives of this study were: (i) to observe the surface morphology, pore structure and chemical compositions of LaFeO_3_-RM; (ii) to evaluate the catalytic activity of the LaFeO_3_-RM/BS system, including the feasibility, mineralization, reusability and stability; and (iii) to identify the main active species and explore the possible mechanism of MB removal in the LaFeO_3_-RM/BS system.

## 2. Material and methods

### 2.1. Chemicals

**Figure 1.**
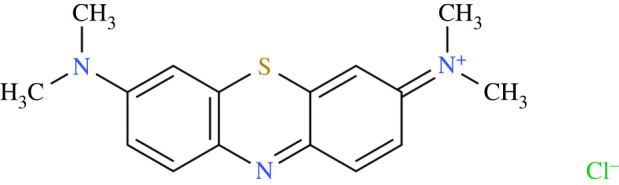
The structure of MB.

A sample of raw RM was obtained from a local RM landfill site in Shandong Province of China. Tert-butyl alcohol (TBA) and methanol (Me) were purchased from Shanghai Aladdin Biochemical Technology Co., Ltd. (Shanghai, China). MB (C_16_H_18_ClN_3_S, 98.5%; the structure is shown in figure 1), anhydrous citric acid (C_6_H_8_O_7_), ferric nitrate non-ahydrate (Fe(NO_3_)_3_·9H_2_O), lanthanum nitrate hexahydrate (La(NO_3_)_3_·6H_2_O), sodium bisulfite (NaHSO_3_), sodium thiosulfate (Na_2_S_2_O_3_), sodium hydroxide (NaOH) and hydrochloric acid (HCl) were purchased from Chengdu Cologne Chemical Reagent Co., Ltd. (Sichuan, China). The reagents were all analytically pure (AR) and were used directly without further purification. Deionized water was used throughout the experiments.

### 2.2. Preparation of LaFeO_3_-RM

LaFeO_3_-RM was synthesized via the sol–gel method. First, the raw RM was dried at 65°C, then it was ground and bagged with a 100-mesh sieve for further use. Then, Fe(NO_3_)_3_·9H_2_O (4.04 g) and La(NO_3_)_3_·6H_2_O (4.33 g) were dissolved into 40 ml deionized water under magnetic stirring. Citric acid (5.76 g) was added after the above solutions were fully mixed together. After that, 4 g of RM powder was added and stirred evenly. Then, the mixed solution was ultrasonically treated for 10 min every 10 min in a water bath of 70°C. After four cycles of ultrasonic treatment, the mixed solution was heated in a water bath of 70°C and mechanically stirred until the sol was formed. Subsequently, the sol was dried overnight at 105°C to obtain the dried red-brown gel. Finally, the dried gel was transferred to a crucible and heated in a muffle furnace at 600°C with a heating rate of 10°C min^−1^ to obtain the LaFeO_3_-RM. For comparison, LaFeO_3_ was also prepared via the same method described above. Also, a sample following the same synthetic method without LaFeO_3_ was denoted as RM-600.

### 2.3. Characterization

The morphology and surface elements of the samples (raw RM, LaFeO_3_ and LaFeO_3_-RM) were analysed by scanning electron microscopy (SEM) and energy-dispersive spectrometer (EDS) equipped with ZEISS Gemini 300. The specific surface area and N_2_ adsorption–desorption isotherms of the samples were determined by the Brunauer–Emmett–Teller (BET) method using Micromeritics ASAP2460. The crystalline phases of the samples were determined by X-ray powder diffractometer (XRD, X’ Pert PRO) using Cu K*α* radiation. The elemental composition and chemical valence state of the samples were determined by X-ray photoelectron spectroscopy (XPS, Thermo Scientific K-Alpha). The functional groups contained in the samples in the range of 4000–400 cm^−1^ were recorded using a Fourier transform infrared spectrometer (FT-IR, Nicolet 670, USA).

### 2.4. Performance evaluation of LaFeO_3_-RM

MB was selected as a model pollutant, and decolorization of MB was tested to evaluate the removal activity of LaFeO_3_-RM. All experiments were carried out in 250 ml beakers at 25 ± 2°C under mechanical stirring with a double-blade impeller (300 r min^−1^) to ensure full blending and sufficient contact with air. The beakers were covered with aluminium foil to avoid light. First, 100 ml MB solution with an initial concentration of 60 mg l^−1^ was prepared, and 10 mmol l^−1^ of BS was added to the solution. Then, its pH was quickly adjusted to 7 with 0.1 M HCl and NaOH. After that, 0.5 g l^−1^ LaFeO_3_-RM was added to the solution to start the reaction (*T* = 0 min). At different time intervals (0, 5, 10, 15, 20, 30, 45 and 60 min), 1.0 ml samples were quickly withdrawn with syringes and filtered through Millipore filters with a pore diameter of 0.45 µm. After that, the samples were transferred into 5 ml centrifuge tubes and 0.1 mol l^−1^ Na_2_S_2_O_3_ was quickly added to quench the reaction.

The effects of different operation parameters on the removal efficiency were also explored, including initial pH (3–11), BS concentration (1–20 mmol l^−1^) and initial MB concentration (10–80 mg l^−1^). Similarly, to further evaluate the active species in the reaction, TBA and methanol (MeOH) with different molar ratios were added to the reaction solution in the quenching experiment. The dissolved oxygen of the system was measured with a dissolved oxygen meter. The chemical oxygen demand (COD) of the MB solution after the reaction was measured using a COD analyser (RB-101H, Guangzhou Ruibin technology, China). Also, the total organic carbon (TOC) was measured using a TOC analyser (LB-CD-800S, LOOBO, China).

Cycle tests were also carried out to investigate the reusability of LaFeO_3_-RM. After each reaction, LaFeO_3_-RM was filtered, recovered and washed with ethanol and water. Then, it was dried in an oven at 65°C for the next cycle. The residual solution was filtered and collected, and the leaching of metal ions was determined by inductively coupled plasma-optical emission spectrometry (ICP-OES, Aglient7800).

### 2.5. Analytic methods

The absorbance of samples was measured by UV-Vis spectrophotometer (Shimadzu UV1780) at the maximum absorption wavelength of 664 nm, and the residual concentration in the MB solution was determined by a standard concentration versus absorbance curve. The removal efficiency of LaFeO_3_-RM was calculated according to equation (2.1):

2.1 $$\mathrm{Removal}\;\mathrm{efficiency}=\left(1-\frac{C_{t}}{C_{0}}\right)\times 100\text{\%}.$$

In addition, the removal process was fitted by the pseudo-first-order kinetic equation (2.2):

2.2 $$\ln\frac{C_{t}}{C_{0}}=-k_{1}t,$$

where *C_t_* represents the concentration of MB at time *t* (mg l^−1^), *C*_0_ represents the initial concentration of MB (mg l^−1^) and *k*_1_ represents the kinetic constant (min^−1^).

All experiments were repeated three times, and the data obtained were averaged for use in figures and tables. The corresponding experimental errors are shown by error bars, which were within ±5% [41].

## 3. Results and discussion

### 3.1. Characterization of LaFeO_3_-RM

#### 3.1.1. Surface morphology and pore structure characteristics

**Figure 2.**
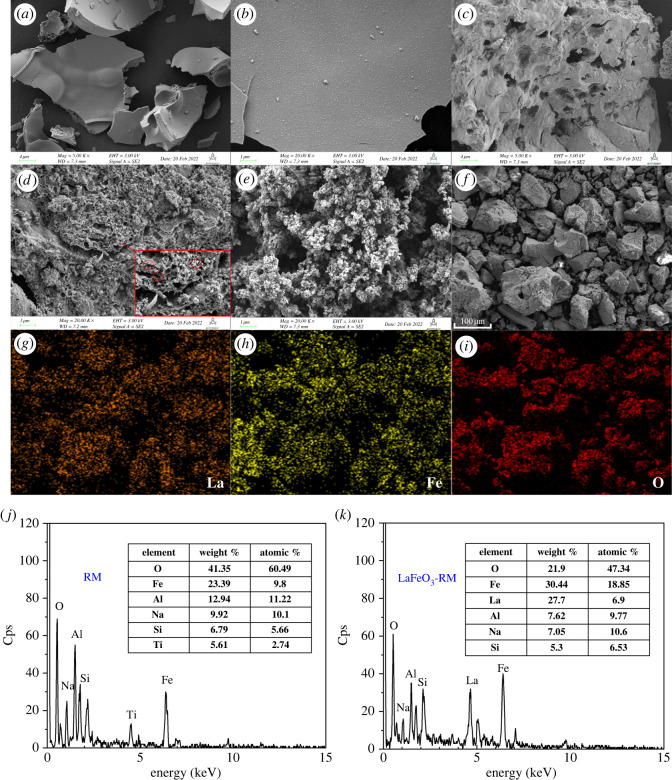
SEM images of LaFeO_3_ (*a*,*b*), LaFeO_3_-RM (*c*,*d*) and raw RM (*e*); element mapping of LaFeO_3_-RM (*f*–*i*); and EDS spectra of the RM (*j*) and LaFeO_3_-RM (*k*).

The surface morphologies of LaFeO_3_, LaFeO_3_-RM and raw RM are shown in figure 2. It can be seen that bulk LaFeO_3_ was mainly in a blocky-flake structure, with a relatively smooth surface and non-porous structure as mentioned in the introduction (figure 2*a*,*b*). However, with RM added for support, the morphology of LaFeO_3_-RM was significantly changed, exhibiting an obvious porous structure and high specific surface area (figure 2*c*,*d*). Some pores with sizes of 100–500 nm can be clearly observed on the surface of LaFeO_3_-RM. The surface morphology of raw RM showed a large number of spherical particles, which aggregate together (figure 2*e*). The element mapping of LaFeO_3_-RM proved the uniform dispersion of LaFeO_3_ on RM, where high contents of La, Fe and O were indicated in the selected area (figure 2*f–i*). The EDS spectrum and chemical composition of RM and LaFeO_3_-RM are summarized in figure 2*j–k*. It shows that the element contents of La and Fe increased significantly in LaFeO_3_-RM. As shown in figure 2*k*, LaFeO_3_-RM was mainly composed of elements of O, Fe, La, Al, Na and Si. The results further confirmed the successful support of LaFeO_3_ on RM.

**Figure 3.**
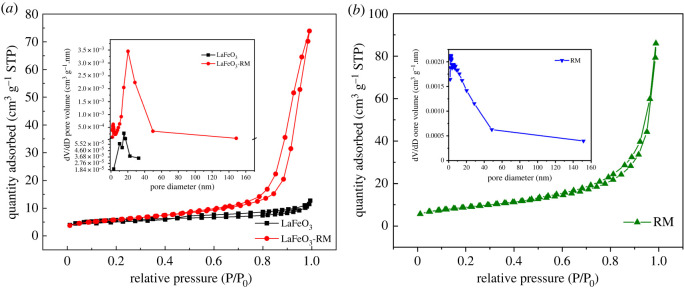
N_2_ adsorption–desorption isotherms and pore size distribution of LaFeO_3_ and LaFeO_3_-RM (*a*) and RM (*b*).

To further investigate the effect of RM support and LaFeO_3_ loading on the structure of LaFeO_3_-RM, N_2_ adsorption–desorption analysis was conducted. As shown in figure 3*a*, the N_2_ adsorption–desorption isotherm of LaFeO_3_-RM was convex downward in the whole pressure range with no inflection point in the curve. According to the International Union of Pure and Applied Chemists classification, the N_2_ adsorption–desorption isotherm of LaFeO_3_-RM belonged to type IV, indicating the porous structure of LaFeO_3_-RM with both mesoporous and microporous characteristics. The nitrogen absorption curve continued increasing with relative pressure (P/P_0_) around 1, suggesting the existence of macroporosity in LaFeO_3_-RM [39]. Also, an H3 hysteresis loop was observed in the isotherm of LaFeO_3_-RM, indicating the presence of narrow-slit pores [42]. However, the isotherm of LaFeO_3_ was of type III, suggesting the non-porous structure of LaFeO_3_. Compared with LaFeO_3_-RM, the adsorption capacity of LaFeO_3_ was significantly lower. Furthermore, the isotherm of LaFeO_3_ crossed with P/P_0_ around 0.4, indicating a small surface area of LaFeO_3_. Figure 3*b* shows that RM exhibited a similar type IV isotherm as LaFeO_3_-RM, indicating that LaFeO_3_ has limited influence on the basic pore structure of RM.

The textural properties of LaFeO_3_, LaFeO_3_-RM and RM are shown in table 1. The specific surface area of LaFeO_3_ was only 9.8216 m^2^ g^−1^, suggesting a small surface area as mentioned in the introduction. However, the specific surface area of LaFeO_3_-RM was twice that of LaFeO_3_, exhibiting a relatively higher specific surface area. The reduction of the specific surface area of RM-supported materials could be due to the blockage of some small pores in the treatment process, which has been reported in previous literature [43]. Also, LaFeO_3_-RM exhibited a high pore volume of 0.114234 cm^3^ g^−1^, which was 10 times higher than the bulk LaFeO_3_, as shown in the pore size distribution curve in figure 3*a*, which indicates that the mesoporosity increased with the addition of LaFeO_3_. Furthermore, the LaFeO_3_-RM showed higher average pore diameters than the RM and bulk LaFeO_3_. The high adsorption capacity of LaFeO_3_-RM could provide a certain space for MB adsorption.

**Table 1.** Textural properties of LaFeO_3_, LaFeO_3_-RM and RM.

sample	*S*_BET_ (m^2^/g)	*V*(cm^3^/g)	*D*_BET_ (nm)	*D*_BJH_ (nm)
LaFeO_3_	9.8216	0.012537	2.9607	17.4172
LaFeO_3_-RM	20.6025	0.114253	15.5045	24.2370
RM	31.1584	0.132480	8.7172	18.6256

#### 3.1.2. Chemical composition characteristics

**Figure 4.**
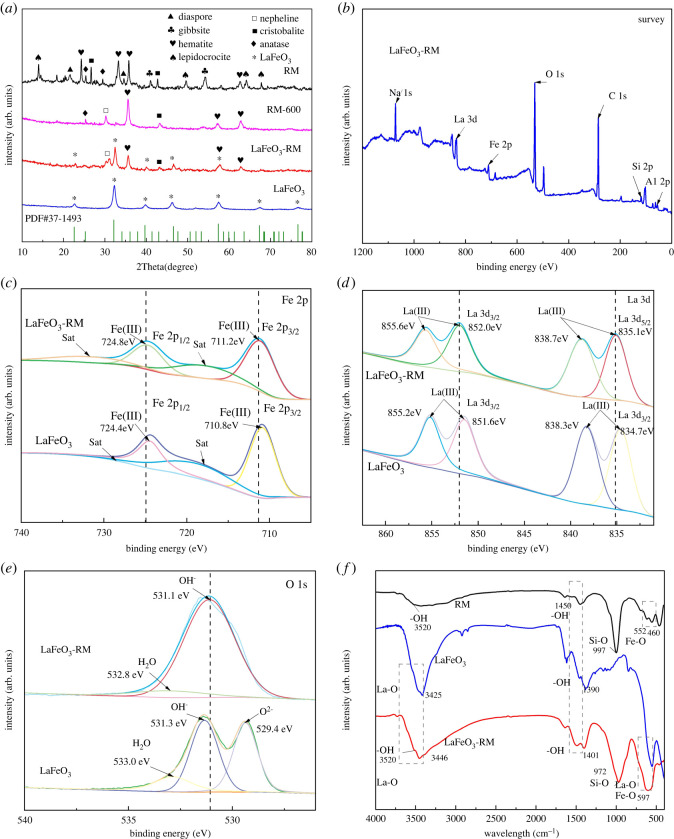
XRD patterns of RM, RM-600, LaFeO_3_ and LaFeO_3_-RM (*a*); XPS survey spectrum of LaFeO_3_-RM (*b*); high-resolution XPS spectrum of LaFeO_3_ and LaFeO_3_-RM for La 3d (*c*), Fe 2p (*d*) and O 1 s (*e*); FTIR spectra of RM, LaFeO_3_ and LaFeO_3_-RM from 400 to 4000 cm^−1^ (*f*).

The XRD patterns of RM, RM-600, LaFeO_3_-RM and LaFeO_3_ are shown in figure 4*a*. The composition of RM was relatively complex with phases determined as gibbsite (α-Al(OH)_3_), hematite (α-Fe_2_O_3_), anatase (TiO_2_), cristobalite (SiO_2_), lepidocrocite (FeO(OH)), nepheline (NaAlSiO_4_) and diaspore (β-AlOOH). As shown in RM-600, the increase of hematite and nepheline with the decomposition of related compounds indicates that the high temperature during the synthesis converted the hydroxide phase into the oxide phase. Also, the diffraction peak corresponding to the Al compound disappeared, indicating the Al compound was decomposed to an amorphous form [44]. After loading LaFeO_3_, the peak intensity of RM was reduced for the blockage of some small pores, as mentioned in the N_2_ adsorption–desorption analysis. As expected, LaFeO_3_-RM showed the main characteristic diffraction peaks of LaFeO_3_ at 2*θ* = 22.6°, 32.3°, 39.8°, 46.4°, 57.4°, 67.4° and 76.8°, which were basically consistent with the diffraction peaks of LaFeO_3_ standard card (PDF# 37-1493). Furthermore, the peak intensity of LaFeO_3_-RM was reduced compared with bulk LaFeO_3_, suggesting highly dispersed LaFeO_3_ on RM as observed in the element mapping of LaFeO_3_-RM.

The elemental composition and chemical valence of LaFeO_3_-RM were determined by XPS analysis, and the results are shown in figure 4*b–e*. Elements such as La, Fe, O, Al, Na, Si, Ti and C were detected on the surface of LaFeO_3_-RM, in which La was derived from LaFeO_3_; Al, Na, Si, Ti were derived from RM; Fe and O were attributed to both RM and LaFeO_3_; and C could be attributed to the amorphous carbon form by the pyrolysis of the citric acid (figure 4*b*). Figure 4*c* shows the high-resolution XPS spectrum of La 3d for LaFeO_3_ and LaFeO_3_-RM. For LaFeO_3_-RM, the peaks at 835.18 eV and 838.88 eV, and 852.08 eV and 855.78 eV corresponded to La 3d_3/2_ and La 3d_5/2_, respectively. The binding energy (BE) difference between La 3d_3/2_ and La 3d_5/2_ was 16.9 eV, indicating the presence of La(III) in LaFeO_3_-RM [45,46]. Compared with LaFeO_3_, the peaks of La 3d_3/2_ and La 3d_5/2_ in LaFeO_3_-RM both shifted to higher BE (+0.4 eV). In the Fe 2p spectrum, for LaFeO_3_-RM, the peaks at 724.8 eV and 724.02 eV corresponded to Fe 2p_1/2_, while the peaks at 711.2 eV corresponded to Fe 2p_3/2_ (figure 4*d*). The BE difference between Fe 2p_3/2_ and Fe 2p_1/2_ was 13.6 eV. Therefore, the two peaks were attributed to Fe(III) [46]. As expected, the peaks of Fe 2p_3/2_ and Fe 2p_1/2_ in LaFeO_3_-RM were also observed shift to higher BE (+0.4 eV) compared with LaFeO_3_, indicating the increase of Fe oxidation capability with RM as a support for LaFeO_3_ [47]. Figure 4*e* shows the spectrum of O 1 s for LaFeO_3_ and LaFeO_3_-RM. For LaFeO_3_, it was observed that the peaks at 529.4 eV, 531.3 eV and 533.0 eV were attributed to lattice oxygen ($O_{2}^{-}$), chemically adsorbed oxygen (OH^−^) and adsorbed water (H_2_O), respectively [48]. For LaFeO_3_-RM, the peaks at 531.0 eV and 532.8 eV were attributed to lattice oxygen and chemically adsorbed oxygen, respectively. As expected, the peaks of O 1 s for LaFeO_3_-RM both shifted to lower BE, further indicating the high oxidation capability of Fe in LaFeO_3_-RM, which could be related to the high oxidation capacity of rich Fe_2_O_3_ in RM [49]. Furthermore, only the peaks of chemically adsorbed oxygen and adsorbed water were observed in LaFeO_3_-RM, while the peak of lattice oxygen disappeared. It was noted that the content of chemically adsorbed oxygen increased significantly from 44.3% to 90.3% with the support of RM. Chemically adsorbed oxygen played an important role in the catalytic performance of the catalyst [32]. Therefore, LaFeO_3_-RM was considered to be an ideal catalyst for its abundant chemically adsorbed oxygen.

The FTIR spectrums of RM, LaFeO_3_ and LaFeO_3_-RM in the range of 400–4000 cm^−1^ are shown in figure 4*f*. The four peaks at 3520 cm^−1^ and 1450 cm^−1^ for RM, 1390 cm^−1^ for LaFeO_3_, and 3520 cm^−1^ and 1401 cm^−1^ for LaFeO_3_-RM belonged to the hydroxyl (OH) groups. The peaks at 997 cm^−1^ for RM and 972 cm^−1^ for LaFeO_3_-RM corresponded to the Si-O groups. The peaks around 3425 cm^−1^ for LaFeO_3_ and 3446 cm^−1^ for LaFeO_3_-RM were attributed to stretching vibrations of La–O. For RM, the peaks at 460 cm^−1^ and 552 cm^−1^ were attributed to the stretching vibrations of Fe-O of Fe_2_O_3_. For LaFeO_3_-RM, the significantly higher intensity peak at 597 cm^−1^ was observed for the stretching vibrations of La-O corresponding to LaFeO_3_ and Fe-O corresponding to both LaFeO_3_ and Fe_2_O_3_ [50]. The result of FTIR analysis further confirmed the successful synthesis of LaFeO_3_-RM, as indicated in the above SEM analysis. It can be seen that, compared with bulk LaFeO_3_, there were abundant oxygen-containing functional groups of -OH and Si-O on LaFeO_3_-RM, which could promote the adsorption of positively charged substances, such as MB molecules, in the aqueous solution process [51].

### 3.2. Methylene blue removal under different systems

**Figure 5.**
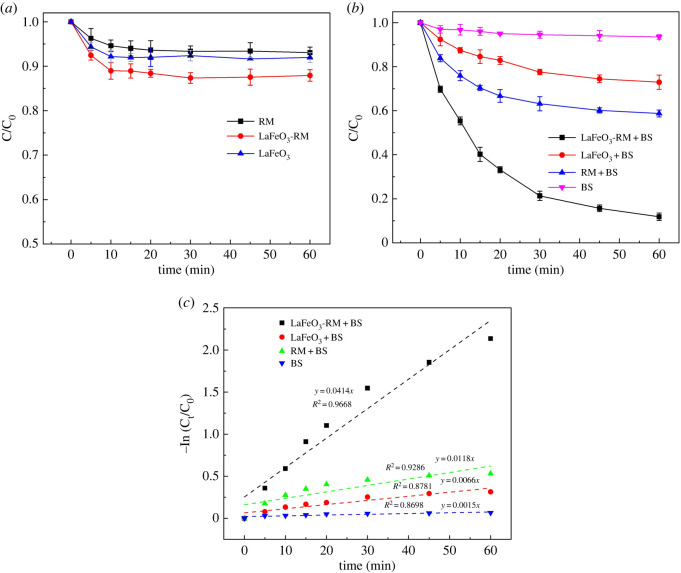
The removal efficiency of MB under different systems: the absence of BS (*a*) and presence of BS (*b*); curves of pseudo-first-order kinetic model on the MB removal by LaFeO_3_-RM, LaFeO_3_, RM and BS (*c*). Experimental conditions: initial pH = 7.0, [Catalyst] = 0.5 g l^−1^, [MB] = 60 mg l^−1^, [BS] = 10 mM l^−1^.

In order to identify the catalytic activity of LaFeO_3_-RM, the removal of MB under different systems was investigated. As shown in figure 5*a*, with the absence of BS, the untreated RM and LaFeO_3_ exhibited a weak adsorption effect on MB (less than 8.5%), while LaFeO_3_-RM had an obvious adsorption effect on MB with an adsorption removal efficiency of 12.10%, probably due to the high specific surface area, increased mesoporosity and abundant surface functional groups of LaFeO_3_-RM. Subsequently, the removal efficiency of MB with the presence of BS was calculated, and the results are shown in figure 5*b*. With the presence of BS alone, the removal efficiency was only 6.55% within 60 min, indicating that BS itself had limited degradation ability for MB, since BS could be oxidized by dissolved oxygen to form sulfate radicals, which could slightly degrade organic pollutants [52]. RM, LaFeO_3_ and LaFeO_3_-RM all showed catalytic effects on MB degradation, with removal efficiencies of 27.09%, 41.32% and 88.19%, respectively. For the RM/BS system, the rich Fe_2_O_3_ in RM provided Fe(III) to activate BS to generate ${\mathrm{SO}}_{4}\cdot^{-}$ for effective degradation of MB, which was proved in previous literature [49]. Figure 5*c* shows that the removal processes followed the pseudo-first-order reaction kinetic model, and the kinetic constant of LaFeO_3_-RM was six times higher than that of LaFeO_3_. Compared with bulk LaFeO_3_, the LaFeO_3_-RM/BS system showed significantly enhanced removal of MB. These results clearly indicate that BS was activated by LaFeO_3_-RM to produce some strong oxidation radicals such as ${\mathrm{SO}}_{4}\cdot^{-}$ and ·OH, which significantly increased the removal of MB [28].

### 3.3. Effect of operating parameters on methylene blue removal

**Figure 6.**
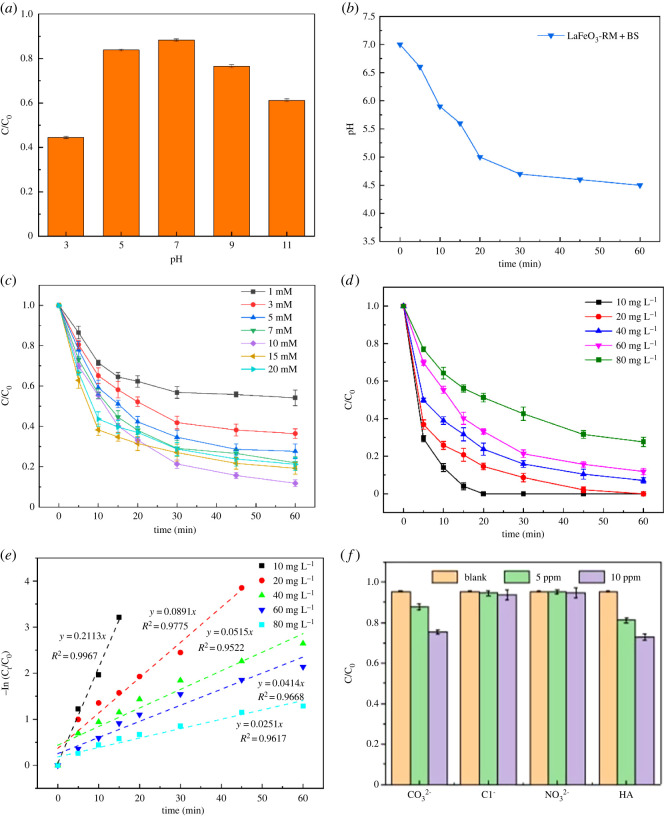
Effects of initial pH (*a*), BS concentration (*c*), initial MB concentration (*d*) and inorganic ions and HA (*f*) on MB removal; changes of solution pH during the reaction (*b*); curves of pseudo-first-order kinetic model on MB removal under different initial MB concentrations (*e*).

To further explore the performance of the LaFeO_3_-RM/BS system, the effects of initial pH, BS concentration and initial MB concentration on MB removal were also investigated, and the results are shown in figure 6.

The initial pH had a certain effect on the removal efficiency of the AOPs system based on sulfate radical (${\mathrm{SO}}_{4}\cdot^{-}$). As shown in figure 6*a*, the removal efficiency of MB decreased sharply under strongly acidic conditions (pH = 3). ${\mathrm{HSO}}_{3}^{-}$ is amphoteric and follows two balance equations:

3.1 $${\mathrm{HSO}}_{3}^{-}\to H^{+}+{\mathrm{SO}}_{3}^{2-},$$

*k*_1_ = 1.02 × 10^−7^

3.2 $${\mathrm{HSO}}_{3}^{-}+H_{2}O\;\to {\mathrm{OH}}^{-}+H_{2}{\mathrm{SO}}_{3},$$

*k*_2_ = 0.65 × 10^−12^

The equilibrium constant of the above equations, *k*_1_ > *k*_2_, indicates that ${\mathrm{HSO}}_{3}^{-}$ mainly exists as ${\mathrm{SO}}_{3}^{2-}$. Therefore, the decrease in removal efficiency under strongly acidic conditions might be caused by the inhibition of BS decomposition, resulting in the reduction of ${\mathrm{SO}}_{3}\cdot^{-}$ (equation (3.8)). The decrease in removal efficiency under alkaline conditions might be attributed to the formation of ·OH by the reaction of ${\mathrm{SO}}_{4}\cdot^{-}$ and water (equation (3.9)). In addition, the standard redox potential of ${\mathrm{SO}}_{4}\cdot^{-}$ was about 3.0 V, while that of ·OH was about 2.8 V. When the initial pH was greater than 9, almost all ${\mathrm{SO}}_{4}\cdot^{-}$ was converted into ·OH (equation (3.4)), leading to a lower degradation efficiency [23]. The equations are as follows:

3.3 $${\mathrm{SO}}_{4}\cdot^{-}+H_{2}O\to \cdot \mathrm{OH}+{\mathrm{SO}}_{4}^{2-}+H^{+}$$

and

3.4 $${\mathrm{SO}}_{4}\cdot^{-}+{\mathrm{OH}}^{-}\to \cdot \mathrm{OH}+{\mathrm{SO}}_{4}^{2-}.$$

Furthermore, the point of zero charges of LaFeO_3_-RM was determined to be 9.1, which could prevent the electrostatic attraction in the system under alkaline conditions [27]. As shown in figure 6*b*, pH decreased significantly in the LaFeO_3_-RM/BS system, indicating the generation of H^+^ in the reaction process. The above results indicate that the LaFeO_3_-RM/BS system could effectively remove MB under neutral and alkaline conditions.

As shown in figure 6*c*, the removal efficiency of MB increased when BS concentration increased from 1 to 10 mM l^−1^. However, with the BS concentration increased from 10 to 20 mM l^−1^, the removal efficiency of MB decreased gradually. When the BS concentration was 20 mM l^−1^, the removal efficiency of MB was only 78.81% within 60 min. It is probable that the reaction between BS and ${\mathrm{SO}}_{4}\cdot^{-}$ (equation (3.5)) and the self-quenching reaction of ${\mathrm{SO}}_{4}\cdot^{-}$ (equation (3.6)) led to the decrease of ${\mathrm{SO}}_{4}\cdot^{-}$ in the system, thus reducing the removal efficiency of MB [53]. Therefore, increasing the BS concentration within a certain range could promote the removal performance of the LaFeO_3_-RM/BS system, and excessive BS concentration could lead to a decline in the removal efficiency. The equations are as follows:

3.5 $${\mathrm{SO}}_{4}\cdot^{-}+{\mathrm{HSO}}_{3}^{-}\to {\mathrm{SO}}_{3}\cdot^{-}+{\mathrm{SO}}_{4}^{2-}+H^{+}$$

and

3.6 $${\mathrm{SO}}_{4}\cdot^{-}+{\mathrm{SO}}_{4}\cdot^{-}\to S_{2}O_{8}^{2-}.$$

The effect of initial MB concentration (10–80 mg l^−1^) on the removal efficiency of MB was also investigated (figure 6*c*). When the BS concentration increased from 10 to 80 mg l^−1^, the removal efficiency of MB showed an obvious decrease from 100% to 72.41%, with pseudo-first-order kinetic constants reduced from 0.2113 to 0.0251 m^−1^, which suggests that the MB removal relied on the active sites on the surface of LaFeO_3_-RM (figure 6*e*). When the concentrations were low, MB molecules could quickly be adsorbed on the spare active sites and degraded. However, with the increased MB concentration, active sites of LaFeO_3_-RM were overloaded so that new MB molecules could only enter the sites after MB molecules that previously occupied the site were degraded. Therefore, in the case of pollutant concentration (60 mg l^−1^), 88.19% of MB was removed in 60 min under neutral conditions (pH = 7), with a small initial dosage of LaFeO_3_-RM (0.5 g/L) and an initial concentration of BS of 10 mM.

### 3.4. Feasibility of the LaFeO_3_-RM/BS system

It is well known that natural water contains a wide range of inorganic ions and humic acids (HA), which may affect the degradation process in AOPs [54]. In order to evaluate the feasibility of the LaFeO_3_-RM/BS system, the effect of inorganic anions (Cl^−^, ${\mathrm{CO}}_{3}^{2-}$ , ${\mathrm{NO}}_{3}^{-}$) and HA on the removal efficiency of MB was investigated. As shown in figure 6*f*, the removal efficiency of MB hardly changed with increased ion concentrations of Cl^−^ and ${\mathrm{NO}}_{3}^{-}$. However, the removal efficiency of MB was significantly decreased when ${\mathrm{CO}}_{3}^{2-}$ concentrations increased from 0 to 10 ppm. This was due to the fact that ${\mathrm{CO}}_{3}^{2-}$ could react with free radicals to form lower active species (${\mathrm{CO}}_{3}^{\cdot -}$ ) (equation (3.7)), resulting in a decrease in MB degradation [54]. In addition, the removal of MB was inhibited with increased HA concentrations because HA could also quench free radicals to slow down the reaction [55]. In conclusion, the LaFeO_3_-RM/BS system still showed a relative high degradation efficiency despite all the effects, indicating its great feasibility for real wastewater.

3.7 $${\mathrm{SO}}_{4}\cdot^{-}+{\mathrm{CO}}_{3}^{2-}\to {\mathrm{SO}}_{4}^{2-}+{\mathrm{CO}}_{3}^{\cdot -}.$$

### 3.5. Mechanisms studies

**Figure 7.**
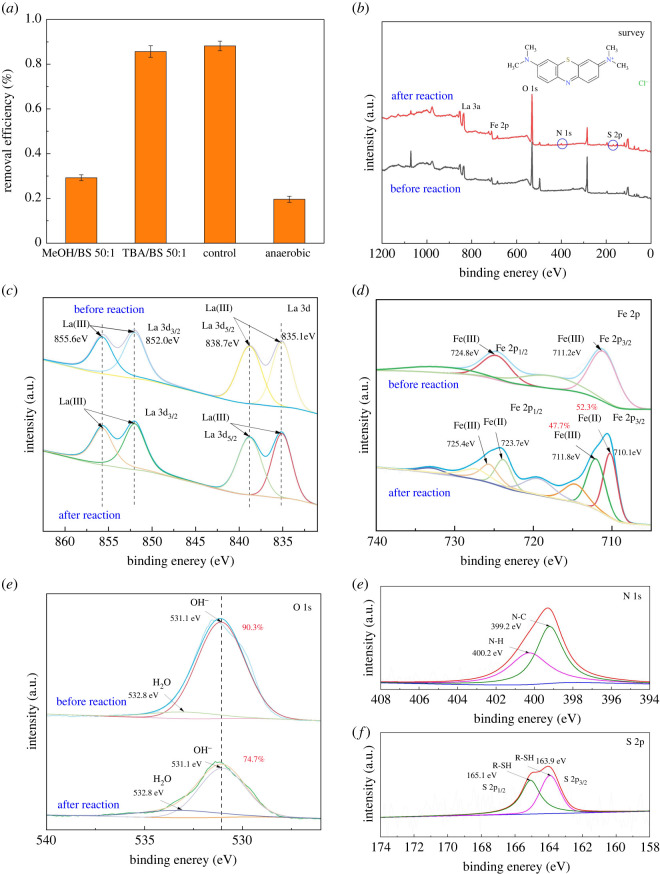
The effect of radical scavengers and O_2_ on MB degradation (*a*); XPS survey spectrum of used LaFeO_3_-RM before and after reaction (*b*); high-resolution XPS spectrum of LaFeO_3_-RM before and after reaction for La 3d (*c*), Fe 2p (*d*), O (*e*), N (*f*) and S (*j*).

As reported, OH· and ${\mathrm{SO}}_{4}\cdot^{-}$ were the main active radicals in the BS activation system. Therefore, to determine the active radicals in the reaction, TBA and methanol (MeOH) were selected as specific radical scavengers for the quenching experiment. MeOH was used to scavenge both OH· and ${\mathrm{SO}}_{4}\cdot^{-}$ (*k*_OH·_ = 0.8–1 × 10^9^ M^−1^ s^−1^, *K*_SO4·−_ = 0.9–1.3 × 10^7^ M^−1^ s^−1^), but TBA was only used to scavenge OH· (*k*_OH·_ = 3.8–7.6 × 10^8^ M^−1^ s^−1^, *K*_SO4·−_ = 4.0–9.1 × 10^5^ M^−1^ s^−1^) [56]. As shown in figure 7*a*, when the molar ratios of MeOH/BS and TBA/BS were 50 : 1, the degradation efficiency of MB decreased by 58.93% and 2.49% within 60 min, respectively, compared with the control group, indicating that the main active radical involved in the system was ${\mathrm{SO}}_{4}\cdot^{-}$.

In addition, the removal efficiency of the LaFeO_3_-RM/BS system under anaerobic conditions using a sealed conical flask was measured under the same experimental conditions. As shown in figure 7*a*, the removal efficiency was only 19.63%, with no obvious catalytic effect observed. By measuring the dissolved oxygen in the reaction process, it was found that the content of dissolved oxygen at 3 min in the normal system (5.4 mg l^−1^ O_2_) was nine times higher than in the sealed system (0.6 mg l^−1^ O_2_). This shows that O_2_ was involved in the reaction, which was ascribed to the abundant dissolved oxygen provided by the mechanical stirring impeller. The result indicates that dissolved oxygen was an essential factor for the generation of ${\mathrm{SO}}_{4}\cdot^{-}$ in the BS activation system [14]. Moreover, the leaching of Fe in the solution after the reaction was measured by ICP-OES. The leaching concentrations of Fe were low (0.042 mg l^−1^), suggesting that the reaction mainly occurred on the surface of LaFeO_3_-RM [29].

In order to further explore the mechanism of the LaFeO_3_-RM/BS system, the XPS analysis of LaFeO_3_-RM after the reaction is shown in figure 7*b–f* [29]. In the La 3d spectrum, the BE and full width at half maximum of peaks of LaFeO_3_-RM remained unchanged after the reaction, suggesting the unchanged valence state of La in the reaction (figure 7*c*). However, in the Fe 2p spectrum shown in figure 7*d*, the peaks at 710.1 eV for Fe 2p_3/2_ and 723.7 eV for Fe 2p_1/2_ were attributed to Fe(II), while the peaks at 711.8 eV for Fe 2p_3/2_ and 725.4 eV for Fe 2p_1/2_ were assigned to Fe(III). The relative amount of Fe(II) increased to 52.3% after reaction, indicating the Fe(III) in LaFeO_3_-RM played a key role in the activation of BS by the redox cycle of Fe(III)/Fe(II) [57–59]. As expected, the chemically adsorbed oxygen (OH^−^) of LaFeO_3_-RM decreased from 90.3% to 74.7% after the reaction, suggesting that chemically adsorbed oxygen directly or indirectly participated in the reaction, which further proved the importance of chemically adsorbed oxygen in the removal process.

Figure 7*f–j* shows that the peaks at 399.2 eV and 400.2 eV were attributed to N-C and N-H, respectively, while the peaks at 163.9 eV for S 2p_3/2_ and 165.1 eV for S 2p_1/2_ were attributed to thiol (R-SH). These bonds of N and S could be derived from the degradation products of the MB molecule (figure 7*b*), indicating that the reaction mainly occurred on the surface of LaFeO_3_-RM with the MB molecule first absorbed on the surface and then degraded.

**Figure 8.**
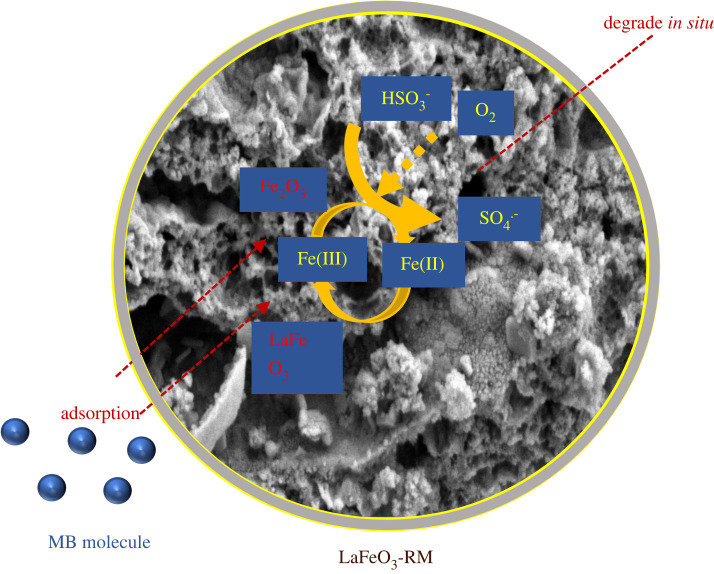
The mechanism of MB removal in the LaFeO_3_-RM/BS system.

According to the experimental results above, we propose the removal mechanism of MB by the LaFeO_3_-RM/BS system as follows (figure 8). First, MB molecules were adsorbed on the surface of LaFeO_3_-RM. Then, BS in the solution aggregated on the surface of LaFeO_3_-RM and reacted with Fe(III) to generate ${\mathrm{SO}}_{3}\cdot^{-}$, while Fe(III) was converted to Fe(II) (equation (3.8)) [60]. Subsequently, ${\mathrm{SO}}_{3}\cdot^{-}$ reacted with O_2_ to generate ${\mathrm{SO}}_{5}\cdot^{-}$ and then reacted with BS to generate ${\mathrm{SO}}_{4}\cdot^{-}$ (equations (3.9–3.10)) [61]. At the same time, Fe(II) reacted with the generated ${\mathrm{SO}}_{5}\cdot^{-}$ to further generate ${\mathrm{SO}}_{4}\cdot^{-}$ and Fe(III) (equation (3.11)), which eliminated the effect of excess Fe(II) on the reaction, and formed the redox cycle of Fe(III)/Fe(II) [62]. Therefore, the MB molecules gathered on the surface were degraded *in situ* by ${\mathrm{SO}}_{4}\cdot^{-}$.

3.8 $$\mathrm{Fe}(\mathrm{III})+{\mathrm{HSO}}_{3}^{-}\to \mathrm{Fe}(\mathrm{II})+H^{+}+{\mathrm{SO}}_{3}\cdot^{-},$$

3.9 $${\mathrm{SO}}_{3}\cdot^{-}+O_{2}\to {\mathrm{SO}}_{5}\cdot^{-},$$

3.10 $${\mathrm{SO}}_{5}\cdot^{-}+{\mathrm{HSO}}_{3}^{-}\to {\mathrm{SO}}_{4}\cdot^{-}+{\mathrm{SO}}_{4}^{2-}+H^{+}$$

3.11 $$\mathrm{and}\;\;2\mathrm{Fe}(\mathrm{II})+{\mathrm{SO}}_{5}\cdot^{-}+H_{2}O\to 2\mathrm{Fe}(\mathrm{III})+{\mathrm{SO}}_{4}\cdot^{-}+2{\mathrm{OH}}^{-}.$$

### 3.6. Mineralization, reusability and stability of LaFeO_3_-RM

The COD and TOC removal of MB were 36.5% and 21.7%, respectively, indicating that MB molecules were partially mineralized. Therefore, intermediates were generated in the degradation process. Some intermediates were measured by GC-MS, and the results are shown in the electronic supplementary material, table S1. According to the generated aromatic products, the chromogenic groups such as -S- and -N(CH_3_)_2_- were destroyed [63]. However, no polycyclic aromatic hydrocarbon oxidation intermediates were generated, which have a high toxicity.

**Figure 9.**
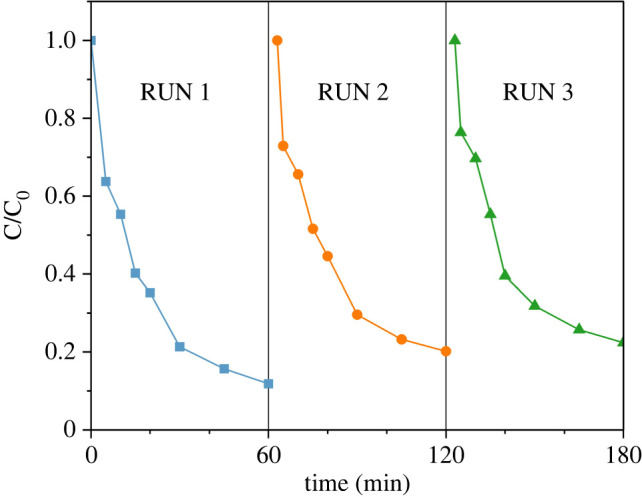
The reusability of the catalyst.

In order to investigate the reusability of LaFeO_3_-RM, it was washed, dried and collected for the next cycle. The results are shown in figure 9. After two and three cycles, the removal efficiency of MB was 79.89% and 77.64%, respectively, which were equivalent to 90.59% and 88.04% of the original removal efficiency. The decrease might be attributed to the residual adsorption of MB molecules on the surface of LaFeO_3_-RM, which blocked the active sites. Therefore, complete regeneration of the catalyst cannot be achieved through a simple washing process.

In addition, the leaching of metal ions in the solution after the reaction was measured by ICP-OES, and the results are shown in table 2.

**Table 2.** Concentrations of heavy metal ions in solution after reaction.

elements	As	Co	Hg	Mn	Ni	La	Cd	Cr
concentrations (mg l^−1^)	ND	ND	ND	0.03 ± 0.02	0.04 ± 0.03	ND	ND	0.05 ± 0.03

It was observed that the leaching concentrations of metal ions were low or undetected (less than 50 ppm), which confirmed the environmental standard formulated by China (Discharge standards of water pollutants for dyeing and finishing of the textile industry (GB4287-2012)), indicating that LaFeO_3_-RM is a promising synergistic catalyst with low environmental risk.

The removal efficiency of supported LaFeO_3_ catalysts used in different heterogeneous Fenton systems for target pollutants in previous literature are shown in table 3. Comparing the result in this work with previous literature, the LaFeO_3_-RM/BS system showed an effective removal of MB, suggesting the superiority of RM as a support for LaFeO_3_. This could be because, in addition to dispersing LaFeO_3_ particles, the porous RM also increased the Fe oxidation activity of LaFeO_3_-RM, which improved the catalytic performance in structure and chemical composition.

**Table 3.** The removal efficiency of the supported LaFeO_3_ catalysts used in different heterogeneous Fenton systems for target pollutants.

ref.	target pollutants		supported LaFeO_3_ catalyst			oxidant			reaction conditions	
	type	[pollutant] (mg L^−1^)	support	dosage (g/L)	type	dosage (mM)	radical	pH	time (min)	degradation rate
this work	MB	60	RM	0.5	BS	10	SO_4_·^−^	7	60	88.19%
[33]	acid orange 7	20	Al_2_O_3_ CeO_2_	0.1	PMS	200	SO_4_·^−^	6.7	120	86.2%
[32]	rhodamine B	9.58	mesoporous silica	2	H_2_O_2_	8.8 × 10^3^	OH·	5.56	60	80%
	MB									60%

## 4. Conclusion

In this study, LaFeO_3_-RM was successfully prepared via ultrasonic-assisted sol–gel method as a synergistic catalyst for MB removal. Characteristics including SEM, BET, XRD, FTIR and XPS of LaFeO_3_-RM revealed that the support of RM significantly improved the catalytic performance of bulk LaFeO_3_ in the porous structure, Fe oxidation activity, oxygen-containing functional groups and chemically adsorbed oxygen (from 44.3% to 90.3%). The effects of different conditions on MB removal, pH, BS dosage and initial MB concentration were investigated. The results showed that the LaFeO_3_-RM/BS system could effectively remove MB under neutral and alkaline conditions. In addition, the enhanced removal mechanism of LaFeO_3_-RM/BS was proposed. MB was removed through the synergistic effect of adsorption and catalysis of LaFeO_3_-RM, with the MB molecule first absorbed on the surface and then degraded by ${\mathrm{SO}}_{4}\cdot^{-}$, which was generated through the activation of BS by LaFeO_3_-RM. Chemically adsorbed oxygen of LaFeO_3_-RM was significantly decreased from 90.3% to 74.7%, suggesting its importance in the removal process. The cycle and leaching tests of LaFeO_3_-RM indicated that LaFeO_3_-RM is an effective and promising synergistic catalyst with repeatability and stability.

## Date accessibility

The data that support the findings of this study are openly available from Dryad Digital Repository: https://doi.org/10.5061/dryad.q573n5tmw [64].

The data are provided in the electronic supplementary material [65].
